# Development of a Caregiver Burden Questionnaire for the Patients with Dementia in Iran

**Published:** 2010

**Authors:** Ibrahim Abdollahpour, Saharnaz Nedjat, Maryam Noroozian, Banafsheh Golestan, Reza Majdzadeh

**Affiliations:** 1 Student in Epidemiology, Epidemiology and Biostatistics Department of Public Health School, Tehran University of Medical Sciences (TUMS), Tehran, Iran; 2 Assistant Professor, Epidemiology and Biostatistics Department of Public Health School, Knowledge Utilization Research Center, TUMS, Tehran, Iran; 3 Associate Professor of Neurology, TUMS, Tehran, Iran; 4 Assistant Professor, Epidemiology and Biostatistics Department of Public Health School, TUMS, Tehran, Iran; 5 Professor, Epidemiology and Biostatistics Department of Public Health School, Knowledge Utilization Research Center, TUMS, Tehran, Iran

**Keywords:** Content validity, Questionnaire development, Care-giver burden, Dementia

## Abstract

**Objectives:**

The purpose of this study was to design a valid questionnaire suitable to the Iranian culture to measure the stress mounted on dementia caregivers.

**Methods:**

In order to design a valid and reliable tool, the stages of content validation were performed as follows: 1- Development: search of relevant electronic databanks and use of experts and caregivers’ opinions to prepare appropriate content, review and correction of the content through consecutive focus group discussions with experts. 2- Judgment Quantification: determination of interrater agreement (IRA), relevancy and clarity of each of the items and the tool as a whole. Reliability was measured with Cronbach’s alpha, and repeatability was measured with intracluster correlation through repeated test-piloting at 2-3 week intervals.

**Results:**

Using a conservative approach, the IRA for the overall relevancy and clarity of the tool was 87.87% and 81.81%, respectively. Through overall agreement (the items that were recognized as appropriate by 100% of the specialists were divided by the total number of items) the overall relevancy of the tool obtained was 98.62%. The overall clarity of the tool was calculated through the mean clarity of the questions and was 99.3%, and eventually its comprehensiveness was 100%. The overall Cronbach’s alpha was 94% and the intracluster correlation that was obtained through comparing the overall score of the questionnaire in the pretest and test phase was 97%.

**Conclusions:**

The new tool has good reliability and validity suitable to Iranian dementia patients and their caregivers’ culture. Researchers can use this tool to monitor the pressure mounted on dementia caregivers and to assess interventions in this group.

## INTRODUCTION

Population aging could be considered as an important consequence of demographic transition, a decline in death rate results in an increase in the proportion of the elderly. On the other hand, a rise in the prevalence of geriatric diseases is a consequence of elderly population growth in all countries which is more remarkable in developing countries like Iran. Dementia is one of the diseases that an increase in its prevalence among the elderly is predictable. Approximately 31 million Iranians are in their middle age now^1^ and they will form the elderly population 20 years later. As dementia prevalence will be highest during that period of life,^2^ we will see a rise in the prevalence of dementia at that time. Dementia is a chronic disease that presents with a series of signs and symptoms such as memory impairment, language disorder, psychological changes, and behavioral impairment.^3^ “Because of the enormous cost of treatment and the burden on the patients and their families resulting from the chronic course and severity of the disease,^4^ dementia is considered as an important public health challenge today”.^5^ This disorder can change the nature of family interactions and increase the risk of psychological problems among them.^6^ While the caregivers influence on treatment outcome and the patients’ needs for the support of families represent the caregivers’ significant and essential role,^7^ the burden imposed on family members of patients with dementia is an important issue which has been neglected. Accepting the role of a caregiver immediately and usually without any preparation,^8^ the nature of the given care, the inordinate length of this responsibility, and the family caregiver’s accountability to the other family members augment the family burden significantly and differentiate them from the other people of the society. Due to stronger family bond among the family members and lack of appropriate social services in elderly care centers, this issue has a higher impact in the societies like Iran. Family and relatives are the main and sometimes the only caregivers for the patients with dementia. A caregiver is defined as a person who devotes about three quarters of his or her daily time to caring for the patients with dementia and often faces trouble and restriction on fulfilling his or her personal, social, and professional responsibilities. A review of relevant literature reveals that the symptoms of depression and anxiety are more common among the caregivers compared to the age-matched control groups.^9^ In contrast to the normal population, caregivers report a lower level of health and life satisfaction too.^6^ In addition, prolonged stress has a negative impact on their physical wellbeing, psychological- mental health, and quality of life resulting in poor quality of patient care, neglect, and even patient abuse.^10^ Therefore, dementia influences not only the patients also their families. The first step in order to identify the factors related to imposed burden on family caregivers taking care of a dementia is the ability of measuring this burden and this would be possible by using a valid and reliable instrument. Since we do not have a proper instrument in Persian language compatible with our patients and caregivers’ culture, this study was carried out to develop an appropriate questionnaire which would be adaptable to the Iranian culture

## METHODS

To design the questionnaire, appropriate content was prepared through literature review and interviews with content experts and lay experts (alert caregivers). Then, the reliability of the instrument was evaluated.

### Literature Review (to prepare the content of the instrument)

To find out the pre-existing instruments, we started this stage by searching different databases (Medline, Google scholar and Scopus). According to the experts’ opinion, the ‘Zarit Burden Interview’ (ZBI) was considered as the main instrument and one of the most commonly used and comprehensive instruments for measuring caregivers’ burden of care for patients with dementia. This questionnaire, which contains 22 questions, has been used in different studies and articles throughout the world.^10^–^13^ The other instruments used in this study were ‘Caring for the Caregiver’^14^ and ’Caregiver Burden Scale.^15^ The initial draft of the questionnaire was prepared after omitting the questions which were common among these three questionnaires.

### Backward and Forward translation

The selected questions were translated to Persian two times by two different translators and after approving the translations, the final Persian version was prepared by the research team. The Persian version was back-translated to English by a person who was proficient in English language and this version was compared to the original questionnaire by a person who knew English as much as a native speaker. Cases of any discrepancies between the concepts of the translated and original version questionnaire were recognized and the necessary changes were made to the Persian translation.

### Content and Lay Experts (alert caregivers)

The initial Persian version of the questionnaire was sent to three neurologists with enough experience of dementia and two psychiatrists to compare it with the original questionnaire and to gain reassurance about its representativeness. To equalize the experts’ conception of content validity indices (relevancy, clarity, and comprehensiveness of the instrument), the definitions of these indices were sent along with the questionnaire. The ability of selected questions to reflect the content was defined as relevancy while the questions lucidity concerning their wording and concept was labeled as clarity, and finally, the instrument’s ability to include all content domains was considered as instrument comprehensiveness. Then, the experts were asked to compare the Persian version with the original one and after reviewing every item, rate its clarity and relevancy from 1 to 4 (1=inappropriate, 2=somewhat appropriate, 3=appropriate, 4=quite appropriate). Moreover, we asked them to:

- Edit the questions in case it was necessary to improve the item’s clarity
- Delete the question(s) which did not have appropriate relevancy from their point of view.
- “Suggest the question(s), adaptable to the Iranian patients and their caregivers’ culture.”

The questions of the questionnaire were sent to five alert caregivers as well. To make the definitions of relevancy, clarity, and instrument comprehensiveness more understandable to caregivers, the definitions sent to the content experts were modified a bit. They were asked to share their opinion on the indices and suggest the questions which they believed were appropriate to the Iranian culture. At this stage, the face validity of the questionnaire was investigated too. The experts’ answers were collected in a 1 to 3-week period. Their opinions about the items wordings, in addition to their suggested questions that were not present in ‘Zarit’, ‘Caring for The Caregivers’, and ‘Caregiver Burden Scale’ were evaluated in several brain-storming sessions in the presence of experts and the team of investigators. In all, twelve brain-storming sessions were held to prepare and approve the questions.

### Pretest

In the summer of 2009, to determine the reliability of the questionnaire and to find out the executive problems, a pretest was carried out on the caregivers referred to ‘Iran Alzheimer Association’. After a 2-3 week interval, the respondents filled in the questionnaire again to assess the repeatability. Caregivers were included in the study only if they had the inclusion criteria of the study which was having a close familial relationship (spouse, daughter, son, grandchild and daughter-in-law) and agreement to participate in the study. The questionnaires were filled in via surveying. The participants were asked to mark the unclear items, if they found any, and explain the ambiguity (unclearness) of each one. Reliability was evaluated in two aspects, repeatability and internal consistency.

### Statistical Analysis

#### A. Agreement on Relevancy and Clarity

A conservative approach was chosen to determine the interrater agreement (IRA) for the instrument relevancy and clarity. In this approach, the number of the questions which all (100%) of the experts (psychiatrists, neurologists, lay experts) chose “quite appropriate” and “appropriate” or “somewhat appropriate𠇜 and “inappropriate” for relevancy (on the other words, the number of questions that all experts agreed on the rate of their appropriateness for relevancy) was divided by the total number of items. IRA for the instrument clarity was calculated exactly by the same method.^16^ The acceptable level (cut-off point) of this index was considered 80% in this study.^17^,^18^

#### B. Clarity and Relevancy for Each Question (item content validity index: I-CVI)

To calculate the clarity of each question, the total number of experts who chose “appropriate” or “quite appropriate” for clarity of each item, was divided by the total number of the experts. Calculation of the relevancy of each item was performed exactly the same way.^16^,^18^–^20^

#### C. Relevancy, Clarity, and Comprehensiveness of the Total Questionnaire (scale content validity index: S-CVI)

The clarity and relevancy of the total instrument are achievable through different methods. In this study, after combining choices “inappropriate” and “somewhat appropriate” and also “appropriate” and “quite appropriate” together, binary choices for each question were computed: appropriate and inappropriate.^16^–^20^ To calculate the relevancy of the total instrument, Scale-Content Validity Index/Universe Agreement approach (S-CVI/UA) was used. In this approach the total number of the questions with appropriate relevancy was divided by the total number of the items.^16^,^18^–^20^ In different articles, the minimum acceptable relevancy for a new instrument has been suggested as 80%.^15^–^18^ Total Clarity of the instrument was computed by dividing the sum of the questions clarity (0-1) or sum of I-CVI_s_ by the total number of questions (mean of the questions clarity or mean of I-CVI_s_). The instrument comprehensiveness was achieved via dividing the number of experts who judged the comprehensiveness of instrument appropriate by the total number of experts. The questionnaire’s total score, which is the indicator of the burden on the caregiver, was calcu-lated by adding the item scores.

#### D. Reliability Indices

Intra-class correlation (ICC) and alpha-Cronbach were used to estimate the reliability and internal consistency, respectively. ICC and alpha-Cronbach’s higher than 0.7 were considered acceptable. The total ICC was obtained by comparing the total scores of the questionnaire in two stages at 2 to 3 weeks interval. The questions, which had reliability problems in either repeatability or internal consistency, were evaluated and proper decisions were made about this group of items by researchers and experts. SPSS (version 15) was used for statistical analysis.

## RESULTS

### 

#### Content Validity

All experts’ (five alert caregivers and five content experts including psychiatrists and neurologists) feedbacks on relevancy, clarity, and comprehensiveness of the 23 questions extracted from the literature review were collected in 1 to 3 weeks (response rate=100%). Besides the 23 items selected from ZBI, Caring for The Caregiver, and Caregiver Burden Scale, the psychiatrists and neurologists suggested 10 more questions which were fitted to characteristics of Iranian Alzheimer patients and their caregivers. These 10 questions were finalized by the team of investigators in brainstorming sessions. After asking the experts for their opinion about the relevancy and clarity of the ten suggested questions, the draft of the questionnaire was prepared which included 33 items. According to the lay and content experts’ questions rating, the IRA used in conservative approach for the clarity and relevancy of these 33 questions were 81.81% and 87.87%, respectively. The relevancy, clarity, and comprehensiveness of the final instrument were 98.18%, 98.78%, and 80%, respectively. Table 1 shows the clarity and relevancy of each item.

**Table 1:** Clarity and relevancy of each question concerning the content and lay experts’ views.

Question	Relevancy	Clarity
1. Do you feel that your patient’s demand for help is more than his/her real needs?	90%	100%
2. Do you feel that you do not have enough time for yourself because of the time you spend on your patient?	100%	100%
3. Do you feel stuck between caring for your patient and fulfilling your other family or work responsibilities?	100%	90%
4. Have you ever felt embarrassed about your patient’s behavior in the presence of your family or friends?	100%	100%
5. Do you feel angry when you are with your patient?	100%	100%
6. Do you feel that your patient’s presence has had a negative effect on your relationships with other family members or friends?	100%	100%
7. Are you afraid of what might happen to your patient in future?	100%	100%
8. Do you feel that your patient’s dependency on you is beyond your capabilities?	100%	100%
9. Do you feel tired when you are with your patient?	100%	100%
10. Do you feel that you are losing your health because of being too involved with your patient’s problems?	100%	100%
11. Do you feel that you do not have as much privacy as you would like because of your patient?	100%	90%
12. Do you feel that your social relationship has suffered because of caring for your patient?	100%	100%
13. Do you feel uncomfortable about inviting your friends to your house because of your patient’s presence?	100%	100%
14. Do you feel that your patient expects you to care for him/her as if you are the only person he/she can rely on?	80%	90%
15. Considering the other expenses you have in life, do you feel that you don’t have enough money for taking care of your patient?	100%	100%
16. Do you feel that you will no longer be able to take care of your patient for a long time?	100%	100%
17. Do you feel that you don’t have enough control over your life since the beginning of your patient’s disease?	100%	100%
18. Do you wish you could leave the care of your patient to someone else?	100%	100%
19. Have you ever been in a situation in which you do not know what the right reaction to your patient’s unusual behavior is?	90%	100%
20. Do you feel that you should pay more attention to your patient?	100%	100%
21. Do you feel that you could have taken better care of your patient?	100%	100%
22. Do you feel that caring for your patient along with other responsibilities has put a heavy burden on you?	100%	100%
*23. Do you feel that your patient needs help 24 hours a day?	100%	100%
24. Do you feel that your patient needs your help to do his/her daily activities?	100%	100%
25. Have you ever felt frustrated over caring for your patient?	80%	90%
26. Do you feel that you have become disappointed, depressed, or gloomy because of coping with your patient’s problems?	100%	100%
27. Do you feel that (in comparison to other family members), most of the burden of taking care of your patient is on you?	100%	100%
28. Has the pressure of taking care of your patient made you abuse him/her and then feel guilty afterwards?	100%	100%
29. Do you feel that taking care of your patient has disturbed your sleep?	100%	100%
30. Do you feel that you are under the pressure of your family members (spouse and children) because of the time and energy you spend for your patient?	100%	100%
31. Have you ever felt that you do not have any help in taking care of your patient?	100%	100%
32. Do you feel that you have lost your personal interests after caring for your patient?	100%	100%
33. On the whole, how burdened do you feel in caring for your patient?	100%	100%

* Questions 23 to 32 (except 25) were suggested by Iranian experts.

#### Reliability

From the 21 participants who filled in the questionnaire at the test stage, only one was not available to refill it (response rate=95%). The participants’ characteristics are showed in Table 2. The mean and median of respondents’ age were 51 (SD=12.5) and the mean and median of their years of education were 9.32 and 12, respectively (SD=5.12).

**Table 2:** Distribution of sex, age, marital, educational and socio-economic status of participants (n=20).

Variable		Mean (SD)	Median	Number	Percentage	Minimum -Maximum
Age (years)		51 (12.5)	51			28-78
The number of years of education		9.32 (5.12)	12			0-14
Home area per capita (square meter)		42	27.5			8-200
Sex	Male			4	%20	
	Female			16	%80	
Marital status	Married			16	%80	
	Single			3	%15	
	Widow			1	%5	
	Divorced			0	%0	
Relationship with the patient	Spouse			6	%30	
	Daughter			7	%35	
	Son			2	%10	
	Grandchild and			5	%25	
	Daughter-in-law					

The average time for completing the questionnaire (by interview) for each person was 14 minutes (range: 10 to 25 minutes). From the respondents’ point of view, there were no unclear question in the questionnaire. As the phrase “spouse and children” in question number 30 (Table 1), “Do you feel that you are under the pressure of your family members (spouse and children) because of the time and energy you spend for your patient?”, did not make sense to single participants, we changed it to “like spouse and children” for this group of interviewees to correct this tiny issue. The mean (SD) of the questionnaire at the test stage was 62 (28.3) and was 63.15 (29.4) at the retest stage. While the reliability of the questionnaire on internal consistency (Cronbach’s alpha) in the test and retest stages were 0.95 and 0.94, respectively, the Intra-Class Correlation (ICC) was estimated at 0.96.

Although the total ICC of the instrument was acceptable, it was not desirable (minimum 70%) for 6 questions. For this reason, and to improve the total ICC of the instrument, these items were discussed in brainstorming sessions by content experts and methodologists and finally the following decisions were made about them: Questions 3 (Do you feel stuck between caring for your patient and fulfilling your other family or work responsibilities?), 13 (Do you feel uncomfortable about inviting your friends to your house because of your patient’s presence?) and 25 (Have you ever felt frustrated over caring for your patient?) had unacceptable ICC, and the content experts and methodologists believed that deletion of these questions would not have any effect on the relevancy (representativeness) and total instrument comprehensiveness. Moreover, these deletions would lead to a shorter time and less expense for completion. So, we decided to delete them. In addition, the content experts believed that the concept of question 22 covered that of question 3 and that its omission would not have any impact on the relevancy and validity of the instrument.

- Although the ICC of questions 19 (Have you ever been in a situation in which you do not know what the right reaction to your patient’s unusual behavior is?), 26 (Do you feel that you have become disappointed, depressed, or gloomy because of coping with your patient’s problems?) and 27 (Do you feel that in comparison to other family members, most of the burden of taking care of your patient is on you?) were lower than the acceptable level (0.497, 0.428, 0.644, respectively), the content experts decided to keep them in the questionnaire because of their high relevancy.
- Questions 20 (Do you feel that you should pay more attention to your patient?) and 21 (Do you feel that you could have taken better care of your patient) were replaced by “Do you feel that you could have taken care of your patient better and more than what you have done so far?“ which includes the concept of both.

After making the abovementioned revisions, the relevancy, clarity, ICC, and Cronbach’s alpha of the total instrument changed to 0.986, 0.993, 0.967, and 0.943, respectively. The changes were implemented according to the content experts’ view and by considering the following two principles arrived at the brain-storming sessions:

- Improving the relevancy and clarity indices of the questions with ICC lower than appropriate level by editing their wording
- Reducing the number of the questions by deleting the items with inappropriate ICC in order to decrease the time (and cost) needed for questionnaire.

Table 3 shows the ICC of each question and Cronbach’s alpha of the total instrument following each question omitted and after making the revisions and preparing the final version of the instrument (at the test stage).

As shown in Table 3, there was no deletion that causes a substantial increase in the Cronbach’s alpha of the total instrument. The used scale for response of each item was a likert scale in which 1, 2, 3, 4 and 5 were respectively assigned to Never, Rarely, Sometimes, Quiet Frequently and Nearly Always. The attainable score ranged from 0 to 116, while a higher score demonstrates a higher burden. Table 4 shows the Cronbach’s alpha, the relevancy, clarity, and total ICC of the questionnaire, before and after making the mentioned changes.

**Table 3:** ICC and Cronbach’s alpha of each question of the final instrument.

Question	ICC of each item	Cronbach’s alpha if item is deleted
1. Do you feel that your patient’s demand for help is more than his/her real needs?	.942	.721
2. Do you feel that you do not have enough time for yourself because of the time you spend on your patient?	.940	.877
3. Have you ever felt embarrassed about your patient’s behavior in the presence of your family or friends?	.943	.716
4. Do you feel angry when you are with (around) your patient?	.941	.897
5. Do you feel that your patient’s presence has had a negative impact on your relationships with other family members or friends?	.939	.917
6. Are you afraid of what might happen to your patient in future?	.943	.723
7. Do you feel that your patient’s dependency on you is beyond (more than) your capabilities?	.943	.853
8. Do you feel tired when you are with your patient?	.939	.74.
9. Do you feel that you are losing your health because of being too involved with your patient’s problems?	.939	.888
10. Do you feel that you do not have as much privacy as you would like because of your patient?	.941	.887
11. Do you feel that your social relationship has suffered because of caring for your patient?	.939	.843
12. Do you feel that your patient expects you to care for him/her as if you are the only person he/she can rely on?	.943	.744
13. Considering the other expenses you have in life, do you feel that you don’t have enough money for taking care of your patient?	.942	.889
14. Do you feel that you will no longer be able to take care of your patient for a long time?	.939	.919
15. Do you feel that you don’t have enough control over your life since the beginning of your patient’s disease?	.938	.887
16. Do you wish you could leave the care of your patient to someone else?	.939	.955
17. Have you ever been in a situation in which you do not know what the right reaction to your patient’s unusual behavior is?	.940	.497
18. Do you feel that you could have taken care of your patient better and more than what you have done so far?”	.946	.610
19 Do you feel that caring for your patient along with other responsibilities has put a heavy burden on you?	.939	.950
20 Do you feel that your patient needs help 24 hours a day?	.940	.891
21. Do you feel that your patient needs your help to do his/her daily activities?	.946	.878
22. Do you feel that you have become disappointed, depressed, or gloomy because of coping with your patient’s problems?	.940	.428
23. Do you feel that (in comparison to other family members), most of the burden of taking care of your patient is on you?	.941	.644
24. Has the pressure of taking care of your patient made you abuse him/her and then feel guilty afterwards?	.940	.798
25. Do you feel that taking care of your patient has disturbed your sleep?	.940	.819
26. Do you feel that you are under the pressure of your family members (spouse and children) because of the time and energy you spend for your patient?	.942	.878
27. Have you ever felt that you do not have any help in taking care of your patient?	.938	.918
28. Do you feel that you have lost your personal interests after caring for your patient?	.940	.898
29. Do you feel that you have lost your personal interests after caring for your patient?	.938	.96

* The Cronbach’s alpha of the total instrument (intra-class correlation) if question was deleted (test stage).

**Table 4:** Statistical findings, before and after making the changes (the final instrument).

Variable	Before changes*		After changes (final instrument)	
Total relevancy	Mean approach	98.18%	Mean approach	98.62 %
Total clarity	Mean approach	98.78%	Mean approach	99.3%
Alpha-Cronbach	Test stage	0.948	Test stage	0.943
	Retest stage	0.944	Retest stage	0.939
Mean (SD)	Test stage	62 (28.3)	Test stage	55.9 (25.6)
	Retest stage	63.15 (29.4)	Retest stage	57.14 (25.9)

Comprehensiveness		80%		100%

Total intra-class correlation		0.958		0.967

* Changes: 3 items (3, 13, 25) were omitted and 2 items (20, 21) were merged.

## DISCUSSION

“Using the content validation process we carried out this study to develop a questionnaire adaptable to Iranian culture for evaluation of “the patient with dementia burden on their family members.” Today, the more quantitative content validation process, using content and lay experts’ views, make this process as a proper tool to design a questionnaire with acceptable relevancy, clarity, and comprehensiveness.^16^,^17^,^19^ Obtaining the experts’ opinion in such type of studies provides the possibility of using proper feedback on the quality of the new instrument. Developing a questionnaire is a process which needs evaluation and revisions, and such revisions make the investigators spend more resources. Using the content validation process may cause an increase in the utilization of resources initially but by decreasing the number of modifications and corrections, and also a reduction in required resources at the correction stages, this process significantly decreases the amount of required resources for the study.^16^ In this research, the content experts recommended one revision phase only. Schutz et al. recommended that IRA is a controlling factor for content validation process.^18^ The acceptable range of IRA has been considered from 70 to 80% in different studies and in this situation, initial modification before content validity analysis is not necessary but its inappropriateness indicates that the questions of the instrument need a fundamental revision.^18^ In this study IRA calculated for relevancy and clarity of the initial version (33-item) was 87.87% and 81.81%, respectively, and the percentages of the final version (29-item) were 86.2% and 89.65%, respectively. These scores represent a high experts’ agreement on the instrument clarity and relevancy. Therefore, since these indices were appropriate, the clarity, relevancy, and comprehensiveness of the instrument (content validity indices) were calculated in the next phase. Mentioning the approach which was used to calculate the CVI is important because significantly different results may be obtained.^19^ The total relevancy of the instrument determined upon mean approach was 98.62%, which indicates that the instrument relevancy was highly appropriate. The range of questions relevancy was between 90 to 100%. This meant that each question of the instrument had suitable relevancy. The minimum acceptable relevancy for a newly developed instrument has been declared 80% in different articles.^16^–^19^ A high relevancy of the instrument shows a selection of representative questions from a list of items with potential for entering the final instrument. The total clarity of the instrument was 99.3%; it was completely appropriate considering the suitable amount (80%) proposed in different articles.^16^–^19^ Proper comprehensiveness for a newly developed instrument has been stated at least 80% in different studies. In this instrument, the total comprehensiveness was 100%, so it could be considered as a perfect and inclusive instrument. Consequently, with respect to the very suitable relevancy, clarity, and comprehensiveness of the newly developed instrument, it could be declared as a valid tool, adaptable to the Iranian culture.

The unacceptable ICC in question number 25, which hints at the caregivers’ disappointment, can be attributed to an ambiguity in “frustration” and its uncommonness in Iranian culture. Due to these reasons we left this question out of the final instrument. The participants’ concern over answering question 13, which asked about the caregivers’ unwillingness in inviting their friends to their houses because of their patient’s presence, might be the reason behind its low ICC. Because the concept of question 22 covered the meaning of question 3, this question’s omission did not influence the relevancy of the instrument. Obtaining the ICC of the question which expresses the concept of questions 20 and 21 (Do you feel that you could have taken care of your patient better and more than what you have ever done?) could be one of the subjects of future studies. The noteworthy point about the questions suggested by the Iranian content and lay experts is the appropriateness of their validity index (relevancy and clarity); none of them was omitted and all of them were included in the final questionnaire. However, the ICC of questions 22 and 23 was not at the appropriate level. Three questions (3, 13 and 25) were left out of the items selected from other questionnaires (Table 1). After implementing the mentioned changes, the time needed for questionnaire completion decreased and the instrument ICC rose from 0.958 to 0.967; the experts believed that those changes did not have any impact on content validity.

In July 2009, after gaining confidence about the questionnaire content validity and to determine its reliability on ICC and internal consistency (using Cronbach’s alpha) we entered the retest stage on the caregivers referred to the Iranian Alzheimer Association in a 2 to 3-week interval. After making the final changes, the total ICC of the instrument was calculated at 0.97; this showed the test repeatability. The ICC in the Brazilian and Chinese versions of the questionnaire was 0.88 and 0.99, respectively.^7^,^8^ The Cronbach’s alpha of the questionnaire was 0.94 in both test and retest stages. In comparison with the ICC of the Brazilian version (0.77 and 0.80 in test and retest stages, respectively) this number showed a high internal consistency of the questionnaire.^7^,^8^ The questionnaire was self-administrated. However, since there aren’t significant differences between verbal and written Persian, and also to prevent selection bias resulting from participants’ illiteracy and to increase their accuracy, the questionnaire could also be filled through an interview (face-to-face or telephone) if the interviewer was trained. However, a face-to-face interview would be more suitable because of a more effective communication and better cooperation between the respondent and the questioner. This finding is comparable to the Brazilian version of ZBI in which the questionnaire can be completed by interview.^8^ However, in the Chinese version the questionnaire was self-administered.^7^ It is possible to find out the existing domains of this questionnaire by carrying out exploratory factor analysis in future studies.

## CONCLUSION

The results of this study confirmed the usefulness of the content validation process to develop valid and reliable content for questionnaires in medical research. Therefore, using the mentioned process, the developed questionnaire has a very appropriate validity and reliability adaptable to Iran’s culture and circumstances. The necessity of precise execution of different stages of content validation, performing required revisions of the generated questions, evaluating the instrument in each revision phase, and returning to the previous stage if it is necessary to determine the validity of a new instrument have been emphasized. Designing “Caregivers Burden of Dementia Patients” questionnaire could be considered as an essential step towards focusing on patients with dementia and their caregivers in Iran because this group of people deserves more consideration and this disease is worthy of more investment.
